# Sector-Specific Patterns of RNFL and Ganglion Cell Complex Thinning Across the Myopia Spectrum: A Cross-Sectional OCTA Study

**DOI:** 10.3390/medicina62061062

**Published:** 2026-05-31

**Authors:** Marija Veselinović, Marija Trenkić, Sonja Cekić, Jasmina Jocić Djordjević, Aleksandar Veselinović

**Affiliations:** 1 Ophthalmology Clinic, University Clinical Center Niš, Boulevard Dr Zorana Đinđića 48, 18000 Niš, Serbia; marija.trenkic@gmail.com (M.T.); sonjaziv@yahoo.com (S.C.); jdjordjevic.jocic@gmail.com (J.J.D.); 2 Faculty of Medicine, University of Niš, Boulevard Dr Zorana Đinđića 81, 18000 Niš, Serbia; 3 Special Hospital for Ophthalmology “Veselinović,” Bulevar Nemanjića 67a, 18000 Niš, Serbia; veselinovic.aleksandar@gmail.com

**Keywords:** myopia, retinal nerve fiber layer, ganglion cell complex, OCTA, axial length, structural biomarker, optical coherence tomography angiography

## Abstract

*Background and Objectives*: Myopia is a rapidly growing global health burden driven primarily by axial elongation, which exerts mechanical stress on the inner retina, leading to progressive thinning of the retinal nerve fiber layer (RNFL) and the ganglion cell complex (GCC). The sector-specific pattern of these changes across the full spectrum of myopia remains incompletely characterized. This study aimed to provide a comprehensive, sector-level analysis of RNFL and GCC changes across four myopia severity grades using optical coherence tomography angiography (OCTA), and to quantify their correlations with axial length (AL) and central foveal thickness (CFD). *Materials and Methods*: A total of 260 eyes of 130 participants were enrolled in a prospective cross-sectional study. Eyes were classified into four groups: emmetropia (EM, n = 74), low myopia (LM, n = 68), moderate myopia (MM, n = 64), and high myopia (HM, n = 54). All participants underwent cycloplegic refraction, AL measurement, and RTVue XR Avanti OCTA imaging. RNFL thickness was assessed across five peripapillary sectors, and GCC thickness across twelve macular zones. Between-group differences were analyzed using one-way ANOVA with Bonferroni post hoc correction or Kruskal–Wallis/Dunn-Bonferroni tests. Pearson correlations were used to assess associations among structural parameters, AL, and CFD. *Results*: Both the RNFL and GCC showed progressive, statistically significant thinning with increasing myopia severity. The superior RNFL was the only peripapillary sector that differentiated EM from LM (*p* = 0.039), with total thinning of 18.8 μm from EM to HM. The paratemporal GCC zone showed the earliest macular structural signal (EM vs. LM, *p* = 0.049). Temporal and nasal RNFL sectors showed relative preservation, with differences restricted to comparisons involving HM. AL correlated negatively with the RNFL and GCC across all sectors (r = −0.46 to −0.71), with the strongest correlation observed for the superior RNFL in HM (r = −0.71, *p* < 0.001). CFD demonstrated progressively stronger coupling with GCC thickness as myopia severity increased, peaking in HM (r = 0.72, *p* < 0.001). *Conclusions*: The RNFL and GCC thinning in myopia follows a progressive, sector-specific pattern driven by axial elongation. The superior RNFL and paratemporal GCC are the earliest structural indicators of inner retinal change, detectable already at the low-myopia grade. These findings support a neural-first model of myopia-related retinal remodeling and advocate for multiparametric, stage-targeted structural monitoring in clinical practice.

## 1. Introduction

Myopia has emerged as one of the most significant global public health challenges of the twenty-first century. A comprehensive meta-analysis of 276 studies encompassing over 5.4 million participants from 50 countries documented a rise in global childhood myopia prevalence from 24.3% in 1990 to 35.8% in 2023, with projections indicating that this figure will exceed 39% by 2050, corresponding to more than 740 million affected children and adolescents worldwide [1]. Landmark projections by Holden et al. estimated that by 2050, nearly 4.8 billion people (49.8% of the global population) will be myopic and approximately 938 million will have high myopia, defined as spherical equivalent ≤ −6.00 diopters or axial length ≥ 26.5 mm [2]. More recent estimates suggest that myopia prevalence has already surpassed 34% among adults globally, with particularly steep increases observed in East Asia and urban environments [3]. This epidemiological trajectory carries profound implications for ocular health, as increasing myopia severity is directly linked to a spectrum of sight-threatening complications that substantially burden healthcare systems worldwide.

Beyond its role as a refractive error, myopia—and high myopia in particular—is a biomechanically driven disease whose primary pathological substrate is progressive axial elongation of the globe [4]. As axial length increases, the posterior segment undergoes geometric distortion: the sclera thins and remodels, the choroid becomes ischemic, and the retina is subjected to mechanical stretching forces that alter both its microstructure and vasculature [5]. These changes confer a substantially elevated risk of severe ocular complications. High myopia is associated with an odds ratio of 845 for myopic maculopathy, 12.6 for retinal detachment, 4.6 for posterior subcapsular cataract, and approximately 3 for open-angle glaucoma relative to emmetropia [6]. Among highly myopic individuals, pathologic myopia—defined as maculopathy equal to or more severe than diffuse chorioretinal atrophy or the presence of a posterior staphyloma—may develop in up to 25% of cases, with half of those individuals progressing to low vision in later life [7]. The growing recognition of myopia as a structural optic neuropathy, rather than a simple refractive condition, has fundamentally changed the approach to its clinical assessment and monitoring.

The inner retina is disproportionately vulnerable to the consequences of axial elongation compared to the outer retinal layers [8]. As the eye elongates, the arcuate nerve fiber bundles originating from the superior and inferior macular regions are subjected to mechanical tension and geometric redistribution, leading to progressive thinning of the retinal nerve fiber layer (RNFL), the layer composed of unmyelinated retinal ganglion cell (RGC) axons converging at the optic disc [9]. Concurrently, the ganglion cell complex (GCC), encompassing the RNFL, ganglion cell layer, and inner plexiform layer, undergoes thinning driven by a combination of mechanical stretch, microvascular insufficiency, and reactive gliosis [8]. Experimental models in marmosets confirm that myopic eye growth primarily affects the inner retina rather than the outer retinal layers, with electroretinographic changes in bipolar, RGC, amacrine, and glial cells potentially preceding structural pathology [10]. At the level of the lamina cribrosa, axial elongation leads to thinning and elongation of this critical structure, impairing axonal transport and increasing the vulnerability of RGC axons to both mechanical damage and ischemia, a mechanism shared with and often clinically indistinguishable from early glaucomatous optic neuropathy [11].

The advent of spectral-domain optical coherence tomography (SD-OCT) and more recently optical coherence tomography angiography (OCTA) has transformed the capacity to quantify these inner retinal structural changes with high precision and reproducibility [12]. SD-OCT allows non-invasive, high-resolution measurement of RNFL and GCC thickness across anatomically defined sectors of the peripapillary and macular regions, providing layer-specific structural information that is critical for monitoring myopia-related neurodegeneration and differentiating it from glaucomatous damage [13]. OCTA extends these capabilities by simultaneously capturing vascular density metrics in the superficial and deep capillary plexuses without the need for contrast agents, enabling a multiparametric assessment of both the structural and vascular dimensions of inner retinal integrity [12]. The RTVue XR Avanti system (Optovue, Inc., Fremont, CA, USA) used in the present study provides validated measurements of RNFL and GCC thickness, along with vascular density parameters, within the same imaging session, making it particularly well suited for a comprehensive, multiparametric evaluation of myopia-related retinal changes.

Despite extensive documentation of structural retinal changes in high myopia, significant gaps remain in the current literature. First, most prior studies have compared only high myopia with emmetropia or have been limited to two or three myopia severity categories, providing an incomplete picture of the progressive and graded nature of inner retinal changes across the full myopia spectrum [14]. Second, most studies have employed peripapillary RNFL or global GCC measurements without detailed quadrant-specific analysis, thereby obscuring the topographic patterns of vulnerability that are most clinically informative [13]. Third, the interrelationship between RNFL thinning, GCC loss, axial length, and central foveal thickness (CFD)—a proxy for the global structural remodeling of the posterior pole—has not been systematically characterized across a graded myopia spectrum in a single cohort with simultaneous structural and vascular OCTA acquisition [15]. Fourth, the question of whether inner retinal structural changes begin at early myopia grades—below the threshold for significant vascular alterations—remains unresolved and has direct implications for the design of early monitoring protocols.

The present study addresses these gaps by providing a comprehensive, quadrant-specific analysis of RNFL and GCC thickness changes across four myopia severity groups—emmetropia (EM), low myopia (LM), moderate myopia (MM), and high myopia (HM)—in a well-characterized cohort of 260 eyes assessed with the RTVue XR Avanti OCTA system. The primary objectives were: (i) to characterize the sector-specific patterns and graded progression of RNFL and GCC thinning across myopia severity; (ii) to identify the earliest structural markers of inner retinal change across the myopia spectrum; (iii) to quantify the correlations between inner retinal structural parameters and axial length and central foveal thickness; and (iv) to contextualize these structural findings within a multiparametric framework that integrates with previously reported vascular OCTA data from the same cohort [15]. By identifying which structural parameters change earliest and most sensitively—and at what stage of myopia—this study aimed to provide actionable evidence to inform the design of targeted, stage-specific structural monitoring strategies for myopic patients.

## 2. Materials and Methods

### 2.1. Study Design, Participants, and Ethics Approval

This was a prospective, cross-sectional, observational study conducted at the Ophthalmology Clinic, University Clinical Center Niš, Niš, Serbia, from 15 September 2020 to 1 March 2021. Clinical data were collected from 260 eyes of 130 consecutively recruited eligible participants. The study was conducted in strict accordance with the ethical principles outlined in the Declaration of Helsinki and received approval from the Ethics Committee of the University Clinical Center Niš (24722/8, date of approval: 8 September 2020) and the Ethics Committee of the Faculty of Medicine, University of Niš. Written informed consent was obtained from all participants prior to enrolment. The present study represented a structural OCTA analysis of inner retinal parameters—specifically RNFL and GCC thickness—from the same cohort previously used to characterize macular vessel density and retinal thickness [15].

All participants underwent a comprehensive ophthalmic evaluation performed by two independent experienced ophthalmologists, which included cycloplegic refraction (Sol. Tropicamide 1%, Speedy-K2 kerato-refractometer; Tomey Corporation, Nagoya, Japan), measurement of intraocular pressure (IOP) by non-contact tonometry, best-corrected visual acuity (BCVA) using Snellen charts, visual field assessment, dilated fundus examination, axial length (AL) measurement using optical biometry (IOLMaster; Carl Zeiss Meditec AG, Jena, Germany), and optical coherence tomography angiography (OCTA) using the RTVue XR Avanti AngioVue system (Optovue, Inc., Fremont, CA, USA). All assessments were conducted under standardized lighting and positioning conditions to minimize measurement variability.

### 2.2. Inclusion and Exclusion Criteria

Inclusion criteria required participants to be aged 18–60 years with BCVA ≥ 1.0 (Snellen), IOP between 10 and 21 mmHg, and axial length between 21.5 and 29.0 mm. Only participants without ocular disease other than refractive error were eligible. Exclusion criteria were defined to exclude potential confounders that could affect retinal microstructure and the integrity of inner retinal layers. A summary of the complete inclusion and exclusion criteria is presented in Table 1. Participant recruitment proceeded consecutively, and each candidate was independently screened by two ophthalmologists to confirm adherence to the eligibility criteria; all discrepancies were resolved by consensus.

Inclusion criteria required participants to be aged 1860 years, with BCVA ≥ 1.0, IOP between 10 and 21 mmHg, axial length between 21.5 and 29.0 mm, and no ocular disease other than refractive error. OCTA scans were required to have SSI > 50 and a quality score ≥ 7/10 with no motion artifacts. Exclusion criteria encompassed: history of neurological or systemic disease (diabetes mellitus, arterial hypertension); prior intraocular surgery or refractive procedure; ocular trauma; media opacity (cataract, corneal pathology); active ocular inflammation or infection; concurrent retinal or optic nerve disease (diabetic retinopathy, glaucoma, macular dystrophies); severe astigmatism > 2.0 D; and poor OCTA image quality due to unstable fixation or blink artifacts.

### 2.3. Myopia Classification

Eyes were classified into four diagnostic groups based on spherical equivalent (SE) values, determined under cycloplegia, and axial length, in accordance with the Expert Consensus on Prevention and Control of High Myopia (2023): emmetropia (EM)—SE between −0.75 D and +0.75 D (n = 74 eyes); low myopia (LM)—SE between −0.75 D and −3.00 D (n = 68 eyes); moderate myopia (MM)—SE between −3.00 D and −6.00 D (n = 64 eyes); and high myopia (HM)—SE ≤ −6.00 D or AL ≥ 26.5 mm (n = 54 eyes).

Group assignment was performed independently by two experienced ophthalmologists, and all discrepancies were resolved by consensus to ensure consistency and accuracy of classification.

### 2.4. Structural Optical Coherence Tomography: RNFL and GCC Acquisition

Structural OCT measurements were obtained using the RTVue XR Avanti Spectral Domain OCT system (Optovue, Inc., Fremont, CA, USA), which acquires high-resolution cross-sectional images of the posterior segment using near-infrared light at 840 nm. Motion correction technology (MCT) was applied throughout all acquisitions to minimize horizontal and vertical motion artifacts. All scans were performed by a single trained operator to ensure consistency, and only scans with a signal strength index (SSI) ≥ 6 were accepted for analysis. In cases of segmentation errors or borderline image quality, the trained operator manually corrected segmentation boundaries.

Retinal nerve fiber layer (RNFL) thickness was measured using the “Optic Disc Cube 4.5 × 4.5 mm” protocol, which provides 200 × 200 A-scan raster coverage of the optic nerve head and peripapillary region. RNFL thickness was defined as the distance from the internal limiting membrane (ILM) to the posterior boundary of the RNFL and was analyzed in five sectors: whole (global average), superior, inferior, nasal, and temporal. Each quadrant spans 90° of the circular peripapillary scan, centered on the optic disc. Automated segmentation was performed using RTVue software, with manual refinement applied as needed.

Ganglion cell complex (GCC) thickness was measured using the “Macular Cube 6 × 6 mm” protocol, with acquisition of 304 B-scans at 20 µm inter-scan spacing, enabling three-dimensional reconstruction of the macular volume. The GCC was defined as the combined thickness of the nerve fiber layer, ganglion cell layer, and inner plexiform layer, measured from the ILM to the outer boundary of the inner plexiform layer. GCC thickness was automatically quantified across twelve anatomical zones centered on the fovea: whole, foveal (1-mm radius), parafoveal (13 mm), and perifoveal (36 mm) regions. The parafoveal and perifoveal regions were each divided into four quadrants: temporal, superior, nasal, and inferior, yielding the following twelve zones: whole, fovea, parafovea, paratemporal, parasuperior, paranasal, parainferior, perifovea, peritemporal, perisuperior, perinasal, and peri-inferior.

Central foveal thickness (CFD) was defined as the minimum retinal thickness at the center of the fovea, automatically extracted from the macular cube scan and verified by visual inspection of B-scans to confirm segmentation accuracy.

### 2.5. Optical Coherence Tomography Angiography (OCTA): Protocol and Parameters

OCTA imaging was performed using the same RTVue XR Avanti system equipped with AngioVue software (Version 2015.1.0.90), which applies a split-spectrum amplitude decorrelation angiography (SSADA) algorithm to detect erythrocyte motion and generate three-dimensional maps of retinal microvasculature without contrast agents. The scan speed was set at 70,000 A-scans per second. Each OCTA acquisition consisted of an X-scan and a Y-scan raster, each lasting approximately 2.9 s, for a total scan time of approximately 6 s. Imaging was centered on the fovea with a 3 × 3 mm^2^ field of view (approximately 10°).

AngioVue software automatically segmented the retinal microvasculature into two vascular plexuses: superficial capillary plexus (SCP), spanning from the ILM to the outer boundary of the ganglion cell layer (GCL), providing superficial vessel density (SVD); and deep capillary plexus (DCP), spanning from the outer boundary of the SCP to the outer boundary of the outer plexiform layer (OPL), providing deep vessel density (DVD).

Additional parameters derived from OCTA in this cohort included retinal thickness (superficial and deep layers), foveal avascular zone (FAZ) area, peripapillary vessel density (PVD), and peripapillary flow index (FI). However, the primary focus of the present analysis was on structural RNFL and GCC thickness parameters. Vascular density findings from this cohort have been reported separately [15].

### 2.6. Image Quality Control and Reproducibility

Rigorous image quality control was applied across both structural OCT and OCTA acquisitions. For structural OCT, only scans with SSI ≥ 6 were accepted. For OCTA, inclusion required SSI > 50 and a quality score of at least 7/10, with absence of motion artifacts, blink artifacts, segmentation errors, or decentration. Three consecutive scans were acquired from each participant for each modality; only the highest-quality scan was selected for analysis.

All scans were independently reviewed by two experienced ophthalmologists with specific expertise in OCTA. In cases of borderline quality, manual correction and realignment of segmentation were performed using RTVue software. In cases of persistent disagreement, a third senior investigator provided the final decision.

Reproducibility was assessed using intraclass correlation coefficients (ICCs). The ICCs for vessel density and retinal thickness measurements were 0.92 and 0.95, respectively, both indicating excellent reproducibility. Bland–Altman plots were constructed to assess the limits of agreement between repeated measurements. The coefficient of variation (CV) was 3.4% for vessel density and 2.8% for retinal thickness, confirming high measurement stability. A sensitivity analysis evaluating the impact of borderline-quality scans on the overall results showed no significant changes in findings.

The same RTVue XR Avanti device and software version (RTVue version 2015.1.0.71) were used for all participants throughout the entire data collection period. Standardized room lighting and participant positioning were maintained across all sessions to reduce external variability.

### 2.7. Statistical Analysis

All statistical analyses were performed using IBM SPSS Statistics version 27.0 (IBM Corp., Armonk, NY, USA), R version 4.3.2 (R Foundation for Statistical Computing, Vienna, Austria; packages: dunn.test, agricolae, rstatix), and Python version 3.11 (Python Software Foundation, Wilmington, DE, USA; libraries: pandas, scipy.stats, matplotlib, seaborn, statsmodels).

Continuous variables were assessed for normality using the Shapiro–Wilk test, supplemented by visual inspection of histograms and Q–Q plots. Homogeneity of variance was evaluated using Levene’s test. When the normality assumption was satisfied, between-group differences were analyzed using one-way analysis of variance (ANOVA), followed by Tukey’s HSD post hoc test for pairwise comparisons. When normality was violated (*p* < 0.05 on Shapiro–Wilk), the Kruskal–Wallis H test was applied, followed by Dunn’s test with Bonferroni correction for pairwise comparisons. Statistical significance was set at *p* < 0.05 for all tests. Quantitative data are reported as means ± standard deviation (SD) unless otherwise specified.

Sex distribution across groups was assessed using the chi-squared (χ^2^) test. The strength of linear associations between RNFL thickness, GCC thickness, axial length (AL), and central foveal thickness (CFD) was evaluated using Pearson correlation coefficients (r), calculated separately for each of the four diagnostic groups and for the overall pooled dataset. Correlation coefficients were interpreted as weak (r = 0.000.30), moderate (r = 0.300.50), strong (r = 0.500.70), and very strong (r > 0.70).

Univariate regression analyses were performed to identify significant predictors of CFD from among RNFL and GCC parameters across the twelve anatomical zones. Variables reaching statistical significance in univariate analysis were subsequently entered into multivariate ordinary least squares (OLS) regression models, constructed separately for each diagnostic group (EM, LM, MM, HM), with CFD as the dependent variable. Prior to model construction, regression assumptions were verified: normality of residuals (Shapiro–Wilk), homoscedasticity (Breusch–Pagan test), absence of multicollinearity (variance inflation factor, VIF < 10), and linearity of associations. Model performance was evaluated using the coefficient of determination (R^2^). Regression equations are reported in the format y = βx + α, and results are visualized as scatterplots with fitted regression lines.

The post hoc statistical power of the study was calculated to confirm the adequacy of the sample size to detect the observed between-group differences in RNFL and GCC thicknesses at α = 0.05. All data were independently double-checked and verified by a third investigator to eliminate transcription or data alignment errors.

## 3. Results

### 3.1. Sociodemographic and Clinical Characteristics

A total of 130 participants (260 eyes) were enrolled across four diagnostic groups. The myopia group comprised 93 patients (48 men, 51.62%; 45 women, 48.38%), with a mean age of 32.65 ± 13.55 years. The control (EM) group included 37 participants (20 men, 54.05%; 17 women, 45.95%), with a mean age of 32.53 ± 13.43 years. No statistically significant differences were found in age (*p* = 0.478) or sex distribution (χ^2^ = 0.003; *p* = 0.955) across the four groups, confirming demographic comparability.

Mean intraocular pressure (IOP) and best-corrected visual acuity (BCVA) were within physiological limits in all groups and did not differ significantly between groups. Axial length (AL) increased progressively and significantly from EM (23.58 ± 0.75 mm) to LM (24.65 ± 0.82 mm), MM (25.88 ± 0.97 mm), and HM (27.91 ± 1.15 mm), with statistically significant differences between all groups involving MM or HM (*p* < 0.05). Notably, the EM–LM difference in AL did not reach statistical significance (*p* > 0.05). Post hoc power analysis (G*Power 3.1; observed difference 3.5 µm; pooled SD 7.5 µm; α = 0.05) yielded a power of 0.61 for the EM–LM whole-GCC comparison, indicating this specific comparison was underpowered. This result should therefore be interpreted as inconclusive rather than as definitive evidence of the absence of structural change at the low myopia stage. Post hoc power analysis (G*Power 3.1) confirmed adequate power (≥0.80) for overall between-group differences in the superior and inferior RNFL sectors and across all GCC zones. For the nasal RNFL overall comparison (power = 0.72), the temporal RNFL overall comparison (power = 0.68), and the EM–LM whole-GCC comparison (power = 0.61), non-significant pairwise results should be interpreted as inconclusive rather than as definitive evidence of the absence of structural difference.

Central foveal thickness (CFD) declined progressively and significantly across the myopia spectrum, from 257.2 ± 11.5 µm in EM to 236.5 ± 13.7 µm in HM (*p* < 0.001 for EM vs. HM). Statistically significant reductions were observed between EM and MM (*p* = 0.041), EM and HM (*p* = 0.002), and LM and HM (*p* = 0.049), whereas the EM–LM difference was not significant. Complete sociodemographic and clinical data are presented in Table 1.

### 3.2. Retinal Nerve Fiber Layer (RNFL) Thickness Across Myopia Groups

**Figure 1:**
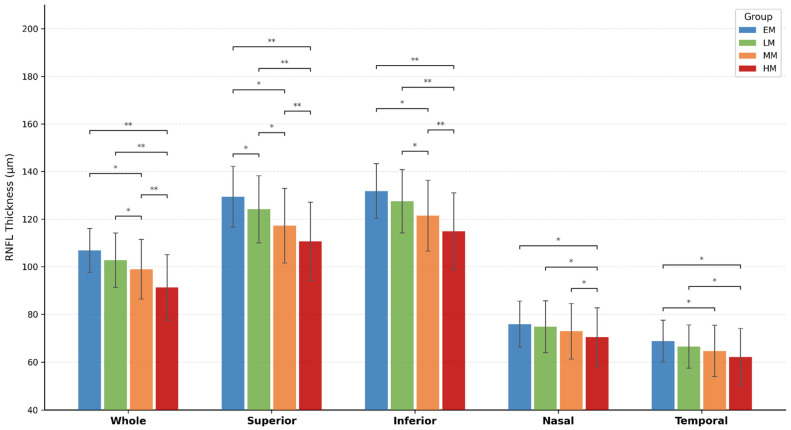
Retinal nerve fiber layer (RNFL) thickness (µm) by sector across four diagnostic groups (EM—emmetropia, LM—low myopia, MM—moderate myopia, HM—high myopia). Values presented as means ± SD. Significance brackets indicate statistically significant pairwise between-group differences in mean RNFL thickness for that sector (* *p* < 0.05; ** *p* < 0.01; one-way ANOVA with Bonferroni post hoc correction).

Statistically significant, severity-dependent thinning of the RNFL was observed across all five examined sectors with increasing myopia grade (Table 2). The grouped bar chart in Figure 1 illustrates a consistent, stepwise reduction in RNFL thickness from EM through LM, MM, and HM across all sectors, with the magnitude of between-group differences increasing with myopia severity.

In the whole RNFL sector, mean thickness decreased from 106.8 ± 9.2 µm in EM to 102.7 ± 11.4 µm in LM, 98.9 ± 12.5 µm in MM, and 91.3 ± 13.8 µm in HM, representing a total reduction of 15.5 µm (14.5%) from EM to HM. Significant pairwise differences were detected between EM and MM (*p* = 0.032), EM and HM (*p* = 0.001), LM and MM (*p* = 0.041), LM and HM (*p* = 0.003), and MM and HM (*p* = 0.002), confirming a broad, severity-dependent pattern of thinning.

The superior RNFL sector demonstrated the earliest and most extensive thinning of all examined zones. Mean thickness declined from 129.4 ± 12.8 µm in EM to 110.6 ± 16.5 µm in HM—a reduction of 18.8 µm (14.5%). Crucially, a statistically significant difference was already detectable between the EM and LM groups (*p* = 0.039), making the superior sector the only RNFL zone to show a significant difference at the earliest transition point in the myopia spectrum. Significant differences were identified in all six pairwise comparisons (EM–LM *p* = 0.039; EM–MM *p* = 0.021; EM–HM *p* = 0.002; LM–MM *p* = 0.033; LM–HM *p* = 0.004; MM–HM *p* = 0.001), underscoring the superior sector as the most sensitive and earliest structural marker of RNFL loss in myopia progression.

The inferior RNFL sector exhibited a pattern closely mirroring that of the superior sector, with mean thickness decreasing from 131.8 ± 11.5 µm in EM to 114.9 ± 16.1 µm in HM (a reduction of 16.9 µm, 12.8%). Statistically significant differences were observed in five of six pairwise comparisons, with the EM–LM comparison being the sole non-significant pair (*p* > 0.05), consistent with the interpretation that inferior RNFL loss lags slightly behind the superior sector in early myopia.

The nasal RNFL sector showed the smallest absolute reduction across the myopia spectrum (75.9 ± 9.6 µm in EM vs. 70.4 ± 12.3 µm in HM; reduction of 5.5 µm, 7.2%), with significant differences confined exclusively to group pairs involving HM (EM–HM *p* = 0.046; LM–HM *p* = 0.039; MM–HM *p* = 0.027). No significant differences were detected in any comparison not involving the HM group, indicating relative preservation of the nasal RNFL at lower myopia grades and confirming this sector’s well-documented resistance to early myopic structural change.

The temporal RNFL sector showed an intermediate pattern, with mean thickness declining from 68.8 ± 8.7 µm in EM to 62.2 ± 11.9 µm in HM (a reduction of 6.6 µm, 9.6%). Significant differences were observed between EM and MM (*p* = 0.041), EM and HM (*p* = 0.029), and LM and HM (*p* = 0.048), but not between MM and HM (*p* > 0.05), suggesting that temporal RNFL thinning decelerates or plateaus at higher myopia grades. This pattern is visible in Figure 1, where the gap between the MM and HM bars in the temporal sector is notably smaller than in the superior and inferior sectors.

Figure 1 provides a comprehensive visual overview of these sector-specific patterns. The error bars reflect standard deviations and illustrate increasing within-group variability at higher myopia grades, particularly in the superior and inferior sectors, consistent with greater heterogeneity of structural damage in high myopia. The significance brackets overlaid on the superior and inferior sectors visually confirm that these are the most statistically robust zones, with the widest span of significant between-group differences.

### 3.3. Ganglion Cell Complex (GCC) Thickness Across Myopia Groups

**Figure 2:**
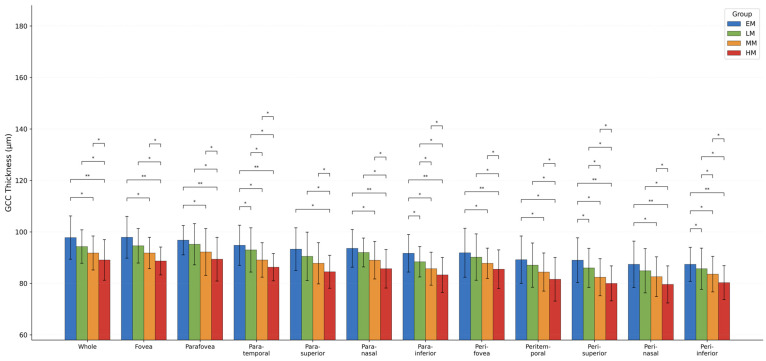
Ganglion cell complex (GCC) thickness (µm) by zone across four diagnostic groups (EM—emmetropia, LM—low myopia, MM—moderate myopia, HM—high myopia). Values presented as means ± SD. Significance brackets indicate statistically significant pairwise between-group differences in mean GCC thickness for that zone (* *p* < 0.05; ** *p* < 0.01; one-way ANOVA with Bonferroni post hoc correction).

GCC thickness showed graded, widespread thinning across all 12 analyzed zones with increasing myopia grade (Table 3). The bar chart in Figure 2 illustrates a consistent reduction in GCC thickness from EM to HM across all zones, with the magnitude of loss varying substantially across anatomical regions. Notably, Figure 2 highlights the paratemporal sector with an EM–LM significance bracket, the only zone in the entire dataset that shows a statistically significant structural difference even at the lowest myopia grade.

Across the entire GCC sector, mean thickness declined from 97.8 ± 8.4 µm in EM to 89.1 ± 7.9 µm in HM (a reduction of 8.7 µm, 8.9%). Significant differences were identified between EM and MM (*p* = 0.032), EM and HM (*p* = 0.002), LM and HM (*p* = 0.041), and MM and HM (*p* = 0.029), while the EM–LM and LM–MM comparisons were not statistically significant.

The foveal GCC zone showed a reduction from 97.9 ± 8.1 µm in EM to 88.7 ± 5.4 µm in HM (reduction of 9.2 µm, 9.4%), with significant differences between EM and MM (*p* = 0.037), EM and HM (*p* = 0.001), LM and HM (*p* = 0.032), and MM and HM (*p* = 0.018). The decreasing standard deviation from EM to HM in the foveal zone—from 8.1 µm to 5.4 µm—suggests progressive convergence of foveal GCC values in high myopia, potentially reflecting a floor effect at the lower boundary of physiological foveal GCC thickness.

The paratemporal GCC zone emerged as the most diagnostically important region in the entire GCC analysis. It was the sole zone to demonstrate a statistically significant difference between the EM and LM groups (*p* = 0.049), indicating that GCC thinning in the paratemporal region begins at the earliest detectable stage of myopia. Mean thickness declined from 94.8 ± 7.8 µm in EM to 93.0 ± 8.6 µm in LM, 89.1 ± 6.7 µm in MM, and 86.3 ± 5.3 µm in HM. Significant differences were detected in five of six pairwise comparisons (all *p* ≤ 0.049), with only the EM–LM comparison being borderline, but still significant (*p* = 0.049). This graded reduction pattern is clearly visible in Figure 2, where the paratemporal bars show the steepest stepwise decline among the parafoveal zones.

The parainferior GCC zone also showed early and severity-dependent thinning. Significant differences were already present between EM and LM (*p* = 0.044) and continued across all group pairs involving MM or HM (*p* = 0.0010.044). Mean thickness decreased from 91.7 ± 7.3 µm in EM to 83.3 ± 6.8 µm in HM—a total reduction of 8.4 µm (9.2%).

Among the perifoveal subregions, the perisuperior zone demonstrated the most progressive and statistically consistent pattern. Significant differences were identified in five of six pairwise comparisons (EM–LM *p* = 0.039; EM–MM *p* = 0.032; EM–HM *p* = 0.001; LM–MM *p* = 0.034; LM–HM *p* = 0.029; MM–HM *p* = 0.016), with mean thickness declining from 89.0 ± 8.7 µm in EM to 80.0 ± 6.8 µm in HM. The peri-inferior zone showed an identical significance pattern (*p* = 0.044 for EM–LM through *p* = 0.001 for EM–HM), with total reduction from 87.4 ± 6.6 µm to 80.3 ± 6.6 µm. These perifoveal findings are highlighted by the significance brackets in Figure 2 overlying the perisuperior and peri-inferior bars, which visually convey the broad and consistent reach of between-group differences in these zones.

The parasuperior and perinasal zones showed intermediate patterns, with significant differences restricted primarily to comparisons involving HM (*p* = 0.031 and *p* = 0.003 for EM–HM, respectively), and the peritemporal zone showed a similar profile. The paranasal zone demonstrated consistent thinning (from 93.6 ± 7.3 µm in EM to 85.7 ± 7.5 µm in HM) with significant differences in most group pairs involving MM or HM, while showing relative preservation at the LM stage.

Taken together, the GCC findings depicted in Figure 2 reveal a consistent topographic pattern: the paratemporal and parainferior parafoveal regions are the earliest and most sensitive indicators of GCC loss, already differentiating low myopia from emmetropia, while the perisuperior and peri-inferior perifoveal regions show the most progressive and widespread between-group differences across the full myopia spectrum. Peripheral GCC zones (perinasal, peritemporal) are relatively preserved at lower myopia grades, with significant thinning emerging primarily in moderate-to-high myopia.

### 3.4. Overview of Pairwise Statistical Differences: RNFL and GCC Heatmap

**Figure 3:**
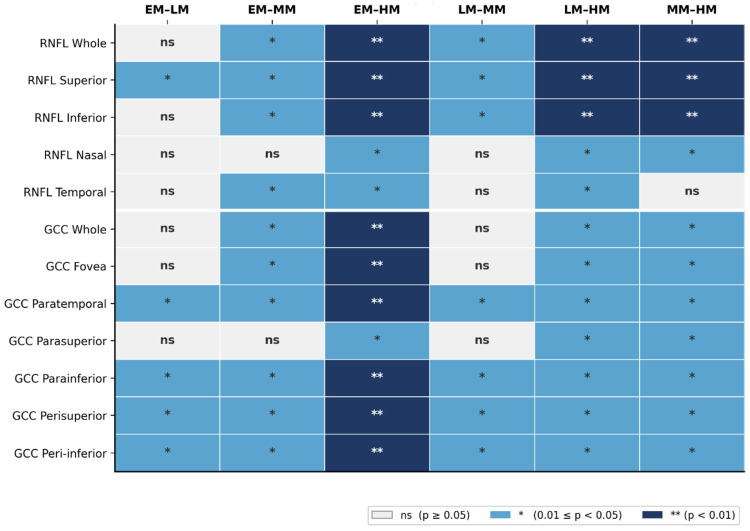
Heatmap of pairwise statistical differences in RNFL and GCC thickness across myopia groups. White cells—not significant (*p* ≥ 0.05); light blue—significant (0.01 ≤ *p* < 0.05); dark blue—highly significant (*p* < 0.01). Asterisks within cells indicate the significance label for each pairwise comparison.

Figure 3 presents a consolidated heatmap of pairwise statistical significance across all RNFL and GCC zones. The three-tier color scheme—white (ns, *p* ≥ 0.05), light blue (significant, 0.01 ≤ *p* < 0.05), and dark blue (highly significant, *p* < 0.01)—provides an immediate visual summary of the pattern and extent of between-group differences across the full structural dataset.

Several key patterns are immediately apparent from Figure 3. First, the RNFL superior sector is the only RNFL zone in which all six pairwise comparisons are statistically significant, making it the most globally sensitive RNFL marker. Second, the GCC paratemporal zone stands out as the only region across both RNFL and GCC where even the EM–LM comparison is significant (light blue cell), confirming its role as the earliest detectable structural indicator in the entire dataset. Third, the columns corresponding to EM–HM and LM–HM comparisons are uniformly dark blue across virtually all zones, reflecting the large magnitude of structural differences that accumulate between emmetropic/low myopic eyes and high myopic eyes.

Fourth, a clear distinction is visible between the RNFL and GCC blocks within the heatmap. The nasal RNFL sector shows the greatest number of white cells (non-significant pairs), confirming its relative resistance across most comparisons. By contrast, all GCC perifoveal zones (perisuperior, peri-inferior) show nearly complete dark blue columns for comparisons involving HM, indicating that perifoveal GCC thinning in high myopia is both universal and highly significant regardless of the comparison group. The heatmap thus provides a rapid, reviewer-friendly synthesis of Table 2 and Table 3, enabling direct identification of the most clinically relevant zones and comparison pairs.

To determine whether the observed GCC thinning reflects genuine structural loss or geometric redistribution of conserved tissue volume over an expanded retinal surface, as postulated by the volume conservation principle of the ocular coats [16,17], and extended to intraretinal layer measurements [18], we performed a geometric correction analysis. Assuming a spherical posterior pole model, the physical area subtended by the fixed 3 × 3 mm^2^ scan field scales as: scan area = 9 mm^2^ × (AL_group/AL_EM)^2^. Under strict volume conservation, the expected GCC thickness in each myopia group would be GCC_expected = GCC_EM × (AL_EM/AL_group)^2^. Applying this formula to the group-mean axial lengths and the emmetropic GCC reference value (97.8 µm; AL_EM = 23.58 mm), the geometry-only model predicts GCC values of 89.49 µm in LM (scan area = 9.84 mm^2^), 81.19 µm in MM (scan area = 10.84 mm^2^), and 69.81 µm in HM (scan area = 12.61 mm^2^). The observed GCC values (94.3 µm in LM, 91.8 µm in MM, 89.1 µm in HM) exceed these geometry-only predictions in all three myopia groups, yielding positive residuals of +4.81 µm in LM, +10.61 µm in MM, and +19.29 µm in HM, indicating partial geometric compensation. Nonetheless, the statistically significant between-group differences (*p* < 0.001, EM vs. HM) confirm that inner retinal thinning beyond pure geometric redistribution occurs in high myopia, consistent with histological evidence of genuine ganglion cell reduction at high axial lengths.

### 3.5. Correlations of RNFL and GCC with Axial Length and Central Foveal Thickness

**Figure 4:**
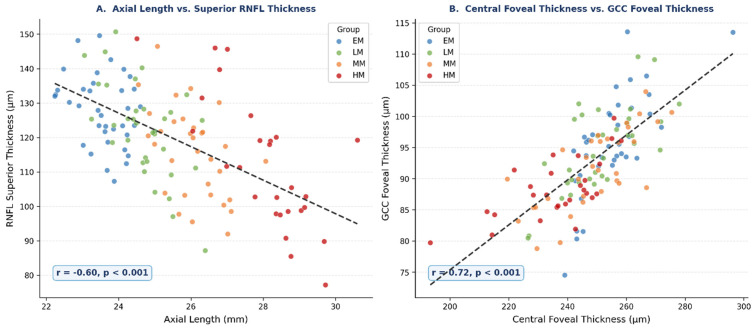
Scatterplots showing (**A**) the negative correlation between axial length and superior RNFL thickness (r = −0.60, *p* < 0.001) and (**B**) the positive correlation between central foveal thickness and GCC foveal thickness (r = 0.72, *p* < 0.001) across all myopia groups. Each point represents an individual eye. Colors indicate emmetropia (EM), low myopia (LM), moderate myopia (MM), and high myopia (HM). Dashed lines represent linear regression fits.

Pearson correlation analysis revealed significant negative associations between axial length (AL) and both RNFL and GCC thickness across all groups, and significant positive associations between central foveal thickness (CFD) and inner retinal layer parameters (Table 4; Figure 4).

AL showed negative correlations with whole RNFL thickness, ranging from r = −0.48 (*p* = 0.021) in EM to r = −0.67 (*p* < 0.001) in HM, with an overall pooled correlation of r = −0.63 (*p* < 0.001). In the emmetropia group, AL–structure correlations were weak to moderate (r = −0.44 to −0.51), reflecting a restricted AL range and dominant physiological variation. By contrast, pooled and HM-group correlations (r = −0.57 to −0.71) reached clinically relevant magnitudes, explaining 32%–50% of the structural variance attributable to AL alone, sufficient to support AL as a meaningful predictor of inner retinal structural status in clinical monitoring. The superior RNFL sector yielded the strongest AL correlations across all groups, reaching r = −0.71 (*p* < 0.001) in the HM group and r = −0.66 (*p* < 0.001) overall, the highest correlation coefficient in the entire analysis. This is illustrated in Figure 4, which shows a scatterplot of AL against superior RNFL thickness for all four groups. The four color-coded point clouds are visually separated along both axes, and the overall regression line (dashed) confirms a strong, consistent negative linear relationship. The progressive leftward and upward shift of each group’s point cloud from HM (red) through MM (orange), LM (green), and EM (blue) reflects the simultaneous increase in AL and RNFL thickness as myopia severity decreases.

AL correlations with GCC thickness were similarly robust. Foveal GCC showed AL correlation coefficients of r = −0.49 in EM (*p* = 0.018) to r = −0.68 in HM (*p* < 0.001), with an overall r = −0.62 (*p* < 0.001). The whole GCC correlated with AL at r = −0.44 in EM to r = −0.62 in HM, overall r = −0.57 (*p* < 0.001). Across all parameters, the strength of AL correlations increased monotonically with myopia severity—from weakest in EM to strongest in HM—indicating a dose-dependent relationship between axial elongation and structural retinal loss.

## 4. Discussion

This study provides a comprehensive, quadrant-specific characterization of RNFL and GCC thickness changes across the full spectrum of myopia severity—from emmetropia through low, moderate, and high myopia—in a well-characterized cohort of 260 eyes imaged with the RTVue XR Avanti OCTA platform. The principal findings are threefold: (1) both RNFL and GCC undergo progressive, sector-specific thinning that scales with myopia severity; (2) the superior RNFL sector and the paratemporal GCC zone are the earliest structural markers of inner retinal loss, with significant differences detectable already at the emmetropia-to-low myopia transition; and (3) axial length and central foveal thickness are strongly and independently correlated with RNFL and GCC thinning, with correlations strengthening progressively at higher myopia grades. These findings are discussed below in the context of the current literature, with emphasis on comparative analysis, biological mechanisms, and clinical implications.

The progressive reduction in RNFL thickness observed across all five sectors in our study is broadly consistent with prior literature documenting RNFL loss in myopic eyes [19,20]. Using Spectralis OCT, Zha et al. reported significant RNFL differences across myopia severity groups in all subfields (*p* < 0.05) [19], and Leung et al. documented that overall RNFL thickness decreased approximately 7 µm per millimeter of axial length using stratus OCT (r = −0.70, *p* < 0.001) [20]. In our study, whole AL–RNFL correlations ranged from r = −0.48 in EM to r = −0.67 in HM, consistent with this magnitude and extending the finding across a graded myopia spectrum using OCTA-derived structural measurements. Similarly, Ganekal et al. reported an average RNFL thinning rate of −5.38 µm per mm of axial length [21], and a cross-sectional Indian study in children found a consistent negative correlation among RNFL thickness, axial length, and increasing myopia severity [22].

The sector-specific hierarchy observed in our data—with the superior and inferior RNFL being most affected, followed by temporal, and nasal being least affected—is in strong agreement with the broader literature. Leung et al. reported that thin RNFL in myopia occurs preferentially at the superior and inferior poles [20], and Sezgin Akcay et al. confirmed, using RTVue SD-OCT, that average, superior, inferior, and nasal RNFL thicknesses were significantly thinner in highly myopic eyes compared to low and moderate myopia, whereas the temporal RNFL showed no significant between-group difference, a finding that matches our temporal results closely [23]. Ganekal et al. similarly found that the RNFL was significantly thinner in high versus low myopia in all quadrants except the temporal quadrant, which paradoxically showed relative preservation or even an increase in thickness at the highest myopia grades, likely attributable to temporal displacement of arcuate RNFL bundles secondary to optic disc tilt [21].

A particularly noteworthy finding in our study is that the superior RNFL sector was the only region that showed a statistically significant difference between the emmetropic and low myopia groups (*p* = 0.039). This is in contrast to Tai et al., who found—after controlling for age, gender, and axial length—that only the HM group differed significantly from EM in the inferior quadrant (*p* = 0.017) [24], and to Sim et al.’s pediatric cohort (PROM-Kids, n = 1000 children), which documented that RNFL thickness decreased in superior, inferior, and nasal quadrants, but increased in the temporal quadrant with higher myopia, with optic disc changes present even in low myopia [25]. The fact that we detect superior RNFL thinning already at the LM stage in an adult cohort may reflect either the cumulative structural burden of sustained low-grade axial elongation, which is distinct from the developmental trajectory in pediatric populations, or the sensitivity advantage of the RTVue OCTA system and our quadrant-based analytical granularity.

Importantly, our finding is also consistent with the anatomical rationale: the superior arcuate fiber bundles originate from the superotemporal macular region and travel a longer arc to the optic disc, making them geometrically more susceptible to the stretching forces of axial elongation than nasal fibers [26]. The differential vulnerability of the superior sector has direct clinical relevance in the context of glaucoma screening in myopic patients, since superior RNFL defects in myopic eyes are known to produce inferior visual field changes that can be misattributed to glaucoma, a risk amplified by the fact that current normative RNFL databases are typically unadjusted for axial length [27].

The relative preservation of the temporal RNFL in our cohort warrants specific discussion. Our data showed no significant difference between MM and HM in the temporal sector, a plateau pattern already recognized in the literature. This has been attributed to the temporalization phenomenon: as axial length increases, the RNFL bundle trajectories shift temporally, effectively redistributing fiber density toward the temporal quadrant and producing an apparent relative thickening or preservation, even as overall RNFL mass is lost [27]. This phenomenon has critical implications for clinical interpretation: a decrease in temporal RNFL thickness in a highly myopic eye may paradoxically signal glaucomatous damage rather than myopic thinning, since myopia tends to preserve or even augment this sector [27]. Our data, showing that the temporal–HM values were not significantly different from MM (*p* > 0.05), corroborate this interpretation and reinforce the need for sector-aware clinical reasoning when interpreting structural OCT in myopic patients.

GCC thinning in our cohort showed a broader and more consistent pattern of between-group differences than RNFL, particularly in the parafoveal subregions. The detection of significant GCC thinning between EM and LM in the paratemporal zone (*p* = 0.049) and parainferior zone (*p* = 0.044) represents the earliest structural signal in our entire dataset. This finding aligns with and extends published data on macular GCC sensitivity in early myopia.

Ucak et al. used OCT-A (Nidek RS-3000) to document that both the superior and inferior GCC were significantly thinner in high myopia than in controls (*p* < 0.001) [28], corroborating the sector-specific vulnerability of the superior and inferior macular inner retina. Our data substantially enrich this picture by showing that GCC thinning begins even before high myopia is established—specifically at the LM grade—in the paratemporal zone, which corresponds anatomically to the projection area of the arcuate macular fiber bundles.

The paratemporal zone’s early vulnerability has a clear mechanistic basis. The temporal macular region is the origin point of the superior and inferior arcuate fiber bundles, whose axons curve around the fovea and converge at the optic disc. With axial elongation, these arcuate bundles are subject to stretch-induced mechanical stress even at modest AL increases, explaining why thinning manifests here before becoming detectable in the broader whole-GCC or perifoveal zones [29]. Małyszczak et al. similarly identified in a Caucasian adult myopic population that GCC parameters showed stronger correlations with axial length than with spherical equivalent, and that these neural deficits were present in both myopic and progressive myopia subgroups, underscoring that axial elongation, rather than refractive error per se, is the primary driver of inner retinal structural loss [30].

Our finding that the perisuperior and peri-inferior GCC zones show the most progressive and statistically universal differences across all group pairs in the perifoveal region is consistent with Wang et al.’s wide-field SS-OCTA study, which reported that GCC thickness and retinal capillary density beyond the 3 × 3 mm^2^ area are more susceptible to axial elongation than central zones, with maximum reduction observed in subregions beyond 6 × 6 mm^2^ [31]. This convergence between our standard 3 × 3 mm^2^ OCTA findings and wide-field OCTA data supports the clinical utility of perifoveal GCC assessment as a sensitive monitoring target for myopia progression.

An important body of literature addresses whether the GCC or peripapillary RNFL is the superior structural biomarker in high myopia, particularly in the context of glaucoma co-diagnosis. Kim et al. demonstrated that the macular GCC had comparable diagnostic ability to the RNFL for detecting glaucomatous changes in highly myopic eyes (AUROC 0.889 vs. 0.825, *p* = 0.442) [32], and Wang et al. reported that GCC global loss volume (GLV) had an AUROC of 0.968 versus 0.855 for the average RNFL in distinguishing high myopic glaucoma from non-glaucoma (*p* < 0.001) [33]. Rezapour et al. further showed that GCIPL and GCC thickness were not significantly associated with axial length in glaucomatous eyes, making them less confounded by myopia-related mechanical distortion than the RNFL, and were therefore more reliable for monitoring glaucoma progression in highly myopic eyes [34].

The geometric correction analysis directly addresses whether observed GCC thinning reflects genuine structural loss or normal geometric redistribution. Szigeti et al., using OCT layer segmentation in 53 healthy eyes, found that total retinal thickness correlated negatively with AL (r = −0.378), whereas no significant correlation was observed for the RNFL or GCC alone, consistent with partial geometric compensation masking layer-specific signals in smaller, less AL-diverse cohorts [18]. In our larger cohort (AL range 21.5–29.0 mm), the positive geometric residuals (+4.81 to +19.29 µm across myopia groups) confirm that GCC volume is partially preserved through redistribution, and the statistically significant between-group differences confirm that pathological structural change superimposes on this geometric variation in high myopia (AL ≥ 26.5 mm).

Taken together, these findings and our data support a dual-parameter strategy for structural monitoring of myopic eyes: the RNFL provides sensitive early detection in the superior sector (as early as LM grade), while the GCC—particularly paratemporal and perifoveal subzones—provides a more myopia-resistant and broadly sensitive readout that extends across the full spectrum from low to high myopia and is less subject to the artifacts, normative database mismatch, and temporalization effects that complicate RNFL interpretation in elongated eyes [27,35].

The negative correlation between axial length and both RNFL and GCC thicknesses in our study strengthened monotonically with increasing myopia severity, reaching peak values in the HM group (r = −0.71 for AL vs. superior RNFL; r = −0.68 for AL vs. foveal GCC). This dose-dependent pattern—correlations weakest in EM, strongest in HM—is consistent across the published literature and supports a model in which axial elongation contributes to inner retinal thinning through two distinct mechanisms: (1) geometric redistribution of partially conserved tissue over an expanded retinal surface, representing normal physiological variation; and (2) excess thinning beyond geometric prediction, most pronounced in high myopia, consistent with pathological structural remodeling as supported by histological evidence.

Takeyama et al. reported GCCAL correlations of r = −0.384 (*p* = 0.001) in a Japanese adult cohort with mean AL of 25.05 mm [36], and Sanepalli et al. confirmed a negative correlation between both RNFL and GCL thickness with increasing axial length and myopia severity, attributing this to scleral stretching transmitted to retinal neural tissue [22]. In our adult population, the correlation magnitudes we report (r = −0.48 to −0.71 for AL–RNFL, pooled r = −0.63; r = −0.44 to −0.68 for AL–GCC fovea, pooled r = −0.62) are somewhat higher than those reported in earlier studies using time-domain OCT with smaller samples, likely reflecting the superior segmentation accuracy of our RTVue XR Avanti platform and the wider AL range in our cohort (21.529.0 mm).

A key mechanistic insight concerns the distinction between axial length and spherical equivalent as predictors of structural loss. Małyszczak et al. found that AL showed stronger correlations with GCC and vascular parameters than SE in their Caucasian cohort [30], a finding corroborated by Ganekal et al., who reported that average RNFL thickness correlated significantly with SE (3.667 µm/diopter) and axial length (−5.38 µm/mm) [21]. An important contributor to the weaker AL–structural correlations in the low-myopia subgroup is the heterogeneity of optical etiology within this subgroup. Barcsay et al. demonstrated that pure corneal origin accounted for 20.9% of low myopic eyes, pure axial origin for 29.2%, and combined etiology for 45.8% [37]. In corneal-origin low myopia, axial length may be within the normal range, attenuating the AL–structural correlation and explaining the modest, underpowered EM–LM structural differences in our cohort. AL-based subgroup stratification is explicitly recommended for future studies. The primacy of AL over SE is mechanistically logical: AL is a direct measure of the geometric distortion imposed on retinal tissue, while SE is a functional refraction metric that reflects optical properties of the whole eye and does not precisely capture the biomechanical state of the posterior segment. Our correlation data, in which AL explains a progressively larger fraction of the variance in RNFL and GCC at higher myopia grades, reinforce the argument that structural retinal monitoring in clinical practice should prioritize AL measurement over SE alone.

The progressive strengthening of AL correlations from EM to HM in our data also suggests a threshold-like acceleration of structural damage at higher axial lengths. This is consistent with Wang et al.’s wide-field OCTA finding that AL > 28 mm represents a critical inflection point at which GCC and capillary density reductions become generalized rather than restricted to peripheral subregions [31], and with Małyszczak et al.’s identification of an axial length of 26.6 mm as a breaking point where the negative FAZ–GCC relationship curve becomes significantly steeper [30]. Although our cohort did not have sufficient power to formally test for AL threshold effects, the progressive strengthening pattern in Table 4 is directionally consistent with a non-linear dose–response model, a hypothesis that warrants formal testing in future studies with larger HM subgroups. Notably, the geometric residual analysis presented in Section 3.3 shows that excess thinning beyond volume-conservation prediction accelerates sharply at AL ≥ 26.5 mm, the threshold defining high myopia in the expert consensus classification, providing indirect support for this boundary as the transition from predominantly physiological geometric variation to pathologically relevant structural change.

The positive correlations between central foveal thickness (CFD) and both RNFL (pooled r = 0.55) and foveal GCC (pooled r = 0.64) documented in our study indicate that foveal thinning co-occurs with inner retinal layer loss in a coordinated rather than independent fashion. The progressive strengthening of the CFD—GCC foveal association from EM (r = 0.61) to HM (r = 0.72) is particularly informative, suggesting that structural coupling between foveal thickness and inner retinal structural loss becomes increasingly tight as high myopia develops.

This coupling is mechanistically interpretable within the framework of progressive axial elongation. As the posterior pole stretches, the mechanical forces acting on the macular region simultaneously thin the outer retinal layers (contributing to CFD reduction) and impose mechanical stress on inner retinal ganglion cells and their axons (contributing to GCC thinning). At low myopia grades, these processes may partially dissociate—outer retinal thinning driven primarily by photoreceptor and RPE layer changes, inner retinal loss driven by fiber bundle stretching—but at high myopia grades, the severity and spatial extent of axial elongation is sufficient to engage both processes simultaneously and in a correlated manner.

The structural coupling model is supported by prior OCTA data from our cohort’s companion publication, which documented strong correlations between deep vessel density (DVD) and retinal thickness in the HM group, and identified DVD and retinal thickness as the most sensitive vascular and structural biomarkers for HM monitoring [15]. The present structural data complement these vascular findings by demonstrating that inner retinal neural architecture follows an analogous coupling trajectory: the stronger the vascular–structure coupling in HM (as reported by the companion study), the stronger the structural—structural coupling we observe between CFD and GCC.

The CFD–RNFL correlation (pooled r = 0.55) is notably lower than the CFD–GCC correlation (pooled r = 0.64), which may reflect the greater susceptibility of RNFL to geometric artifacts in myopic eyes—including the lateral displacement of RNFL peaks due to optic disc tilt—that reduces the fidelity of the RNFL as a fovea-anchored structural biomarker. The GCC, measured in the macular area centered on the fovea, avoids these peripapillary geometric distortions and may therefore provide a more coherent representation of inner retinal architecture in relation to foveal structural status. This differential is clinically relevant: in highly myopic eyes where RNFL measurements are most susceptible to artifacts and normative database misclassification, CFD–GCC correlations may serve as an internal consistency check: if foveal thinning is present and CFD–GCC coupling is maintained, the GCC finding is likely to be authentic rather than artifactual [32].

A recurring theme in the structural OCT literature is the difficulty of distinguishing myopia-related RNFL and GCC thinning from early glaucomatous damage, which follows overlapping anatomical patterns [27]. Our data contribute to this differential in several specific ways.

First, the consistent preservation of the nasal RNFL sector across most pairwise comparisons in our cohort (significant differences only in pairs involving HM) supports using the nasal RNFL as a reference zone: a disproportionate reduction in the nasal RNFL relative to superior/inferior loss would be atypical for pure myopic thinning and more suggestive of concurrent glaucomatous damage. This is consistent with the observation in the glaucoma literature that the nasal RNFL tends to be spared in typical glaucomatous arcuate damage, which preferentially affects the superotemporal and inferotemporal sectors [38].

Second, the temporal RNFL preservation pattern—with no significant MM–HM difference in our cohort—provides a specific differentiating signal: in myopic eyes, temporal RNFL is expected to be relatively preserved or even augmented by the temporalization phenomenon, and a significant temporal RNFL reduction in a high myope should raise the clinical suspicion for glaucoma superimposed on myopic thinning [27]. Markeviciute et al. confirmed this in a cohort of 42 participants with open-angle glaucoma, high myopia, or both, finding that temporal quadrant RNFL was highest in the high myopia-only group, and that temporal RNFL reduction, together with macular RNFL thinning and inferior macular vessel density reduction, were the most informative parameters for diagnosing glaucoma superimposed on high myopia [39].

Third, the GCC superiority over RNFL in high myopia for structural monitoring purposes—documented in prior studies and supported by our CFD–GCC correlation data—has been confirmed by systematic review, which concluded that inferotemporal GCIPL and GCC thickness are the best macular OCT parameters for detecting glaucoma in highly myopic eyes, with GCIPL hemifield asymmetry analysis being particularly effective in high myopia with tessellated fundus where peripapillary RNFL defects cannot be clearly delineated [35]. Our sector-specific GCC data identify the paratemporal and peri-inferior zones as the earliest sites of thinning, providing a topographic target for asymmetry-based glaucoma screening in myopic patients.

Fourth, the progressive normalization of OCT databases for axial length is a critical unresolved clinical challenge. A recent ARVO 2025 presentation from a Colombian cohort found that existing normative RNFL databases are sufficient for high myopia, but insufficient for mild-to-moderate myopia in Latin populations, leading to false-positive glaucoma classifications at lower myopia grades [40]. Our data, demonstrating significant RNFL thinning at the LM grade in the superior sector, reinforce the need for myopia-specific, axial-length-adjusted normative databases across the full myopia spectrum, not only for severe cases.

A central strength of this study is that it derives from the same cohort as a previously published OCTA analysis of macular vessel density and retinal thickness in myopia [15], enabling direct cross-referencing of structural and vascular parameters within the same eyes. The companion study identified deep vessel density (DVD) and retinal thickness as the most sensitive OCTA-derived biomarkers for high myopia, with DVD reductions most pronounced in the peritemporal and peri-inferior sectors and becoming the dominant findings only from moderate myopia onward. The current structural analysis reveals a complementary and temporally preceding pattern: RNFL and GCC thinning are detectable in the superior and paratemporal sectors already at the LM grade, before vascular density changes reach statistical significance.

This temporal dissociation—structural neural changes preceding detectable vascular alterations—has important mechanistic and clinical implications. From a mechanistic perspective, it suggests that the primary insult of axial elongation acts first on neural tissue (mechanical stretching of RNFL bundles and displacement of ganglion cell axons), with vascular remodeling representing a secondary adaptive or degenerative response to the neural and metabolic demand changes that follow. This is broadly consistent with the vascular hypothesis of myopia, which posits that reduced retinal blood flow and microvascular density in high myopia are partly a consequence of reduced metabolic demand from thinned inner retinal tissue, rather than a primary cause [27].

From a clinical perspective, the integration of structural (RNFL, GCC) and vascular (SVD, DVD) OCTA parameters from the same imaging session offers a practical multiparametric monitoring framework for myopic patients. At the low-myopia stage, the superior RNFL and paratemporal GCC provide the earliest structural signal and vascular parameters are not yet significantly altered. Monitoring strategies should prioritize structural OCTA. At the moderate myopia stage, GCC thinning becomes widespread across parafoveal and perifoveal zones, DVD begins to show significant differences in specific sectors. A combined structural + deep vascular assessment is optimal. At the high-myopia stage, the RNFL and GCC show extensive, significant thinning across nearly all zones, DVD reduction is generalized and correlated with structural parameters, and CFD–GCC coupling is strongest. The full multiparametric profile (RNFL, GCC, DVD, SVD, CFD, AL) should be documented and tracked longitudinally, with special attention to temporal RNFL and GCC asymmetry as potential indicators of superimposed glaucomatous damage.

Lai et al. provided longitudinal OCTA evidence supporting this framework, demonstrating in a cohort of 61 normal-tension glaucoma eyes with and without high myopia that RNFL thickness loss and vascular density decline occurred simultaneously, but that AL was independently and negatively correlated with the rate of both RNFL loss and visual field progression in highly myopic NTG eyes [41]. This longitudinal confirmation of the AL-mediated structural progression trajectory reinforces the clinical value of serial AL monitoring as an independent risk stratifier, complementing the structural and vascular OCTA parameters described in the present and companion studies.

Several limitations of this study should be acknowledged. First, the cross-sectional design precludes establishing temporal sequences among axial elongation, structural thinning, and vascular remodeling. Longitudinal data are needed to confirm whether superior RNFL and paratemporal GCC thinning genuinely precede vascular alterations in the natural history of myopia progression. Second, our study was conducted at a single clinical center in Serbia, with a predominantly Caucasian adult population. Published data suggest meaningful ethnic differences in RNFL normative values (Caucasians tend to have slightly thinner RNFLs than Hispanics or Asians), and the generalizability of our quadrant-specific findings to pediatric or Asian populations, where myopia prevalence and axial elongation rates are substantially higher, requires cautious interpretation [26].

Third, the RTVue XR Avanti system, while providing excellent reproducibility (ICC 0.920.95 in our cohort), relies on segmentation algorithms that may be less accurate in eyes with the most extreme axial lengths, tessellated fundus, or early posterior staphyloma. The potential for segmentation artifacts in HM eyes, particularly for RNFL measurements at the optic disc boundary, cannot be entirely ruled out despite our manual quality-control protocol.

Fourth, we did not formally assess participants for subclinical glaucoma using visual field testing or optic disc stereophotography, and the overlap between myopia-related and early glaucomatous structural thinning in the superior and inferior RNFL sectors cannot be definitively excluded. Future studies in this cohort should incorporate visual field assessment and disc hemorrhage documentation to formally adjudicate structural changes attributable to myopia versus those potentially representing preperimetric glaucoma.

Fifth, the sample size in the HM subgroup (n = 54 eyes) limits statistical power for subgroup analyses within the high-myopia category, such as stratification by AL above or below 28 mm, a threshold proposed as clinically meaningful in prior wide-field OCTA studies [31]. Expanding the HM subgroup in future longitudinal follow-up would enable formal threshold analysis and assessment of AL-specific structural trajectories within the high-myopia range. Furthermore, using spherical equivalents for subgroup classification in the low- and moderate-myopia grades may have introduced axial-length heterogeneity within these groups, particularly because some low-myopia cases are of corneal rather than axial origin [37]. Future studies should adopt AL-based stratification to reduce this variance and improve the sensitivity of structural comparisons across myopia grades.

## 5. Conclusions

This study provides a comprehensive, quadrant-specific characterization of RNFL and GCC changes across the full spectrum of myopia severity using OCTA. Both structural parameters undergo progressive, sector-specific thinning that scales with myopia severity and is driven by axial elongation. The total reduction in the superior RNFL from emmetropia to high myopia reached 18.8 µm (14.5%), while GCC paratemporal thickness declined by 8.5 µm (9.0%) across the same range, reductions that are clinically significant and detectable with current imaging technology.

The superior RNFL sector emerges as the earliest structural indicator of inner retinal loss in myopia, showing significant thinning already at the emmetropia-to-low myopia transition (*p* = 0.039), a finding not previously documented in an adult cohort with simultaneous structural and vascular OCTA acquisition. At the macular level, the paratemporal GCC zone represents the earliest structural signal (*p* = 0.049), making it the single most sensitive quadrant-level biomarker in the dataset. Integrated reading of the heatmap, sector-specific bar charts, and correlation scatterplots reveals two clinically distinct topographic patterns: a peripapillary pattern dominated by a superior > inferior > temporal > nasal RNFL hierarchy, consistent with the anatomical distribution of arcuate fiber bundles, and a macular pattern centered on paratemporal and peri-inferior GCC vulnerability, reflecting the spatial projection of macular arcuate fibers. The relative preservation of temporal and nasal RNFL across most myopia grades provides a useful reference frame for distinguishing myopia-related thinning from glaucomatous damage in highly myopic eyes.

Axial length is the primary, dose-dependent driver of both RNFL and GCC loss, with correlations increasing monotonically across myopia grades and reaching r = −0.71 between AL and superior RNFL in the high-myopia group. This underscores the primacy of AL monitoring over spherical equivalent alone in clinical practice. Central foveal thickness demonstrates progressively tighter coupling with GCC foveal thickness as myopia worsens (r = 0.72 in high myopia), reflecting coordinated remodeling of both outer and inner macular layers under the mechanical forces of sustained axial elongation.

When combined with the vascular OCTA data from the companion publication, which identified deep vessel density and retinal thickness as the earliest vascular biomarkers—detectable only from moderate myopia onward—the present structural findings establish that neural structural changes precede vascular alterations in myopia progression. This temporal dissociation has direct implications for surveillance strategy: structural assessment of the superior RNFL and paratemporal GCC should be prioritized even at low myopia grades, while vascular monitoring becomes increasingly relevant as disease advances. These conclusions support implementing a multiparametric, stage-specific OCTA monitoring approach that integrates RNFL, GCC, axial length, and central foveal thickness into a coordinated panel of structural biomarkers.

## Figures and Tables

**Table 1:** Sociodemographic and clinical characteristics of the four diagnostic groups.

Characteristic	EM (n = 74)	LM (n = 68)	MM (n = 64)	HM (n = 54)	*p*-Value
Male/female (n)	20/17	26/18	33/15	28/26	0.955
Age (years)	32.53 ± 13.43	32.65 ± 13.55	32.65 ± 13.55	32.65 ± 13.55	0.478
IOP (mmHg)	15.4 ± 2.3	16.2 ± 2.1	16.1 ± 2.2	16.3 ± 2.0	ns
BCVA	0.98 ± 0.03	0.95 ± 0.05	0.95 ± 0.05	0.94 ± 0.06	ns
SE (diopters)	0.00 ± 0.50	−1.87 ± 0.55	−4.52 ± 0.83	−7.91 ± 1.44	<0.001
AL (mm)	23.58 ± 0.75	24.65 ± 0.82	25.88 ± 0.97	27.91 ± 1.15	<0.001
CFD (µm)	257.2 ± 11.5	251.4 ± 12.2	248.6 ± 13.0	236.5 ± 13.7	<0.001

EM—emmetropia; LM—low myopia; MM—moderate myopia; HM—high myopia; IOP—intraocular pressure; BCVA—best-corrected visual acuity; SE—spherical equivalent; AL—axial length; CFD—central foveal thickness; ns—not significant. Values expressed as means ± SD unless otherwise indicated.

**Table 2:** Mean retinal nerve fiber layer (RNFL) thickness (µm) by sector across four diagnostic groups.

Zone	EM	LM	MM	HM	EM–LM	EM–MM	EM–HM	LM–MM	LM–HM	MM–HM
Whole	106.8 ± 9.2	102.7 ± 11.4	98.9 ± 12.5	91.3 ± 13.8	ns	0.032	0.001	0.041	0.003	0.002
Superior	129.4 ± 12.8	124.1 ± 14.1	117.2 ± 15.7	110.6 ± 16.5	0.039	0.021	0.002	0.033	0.004	0.001
Inferior	131.8 ± 11.5	127.5 ± 13.3	121.4 ± 14.9	114.9 ± 16.1	ns	0.029	0.004	0.041	0.003	0.001
Nasal	75.9 ± 9.6	74.8 ± 10.8	72.9 ± 11.6	70.4 ± 12.3	ns	ns	0.046	ns	0.039	0.027
Temporal	68.8 ± 8.7	66.5 ± 9.0	64.7 ± 10.7	62.2 ± 11.9	ns	0.041	0.029	ns	0.048	ns

EM—emmetropia; LM—low myopia; MM—moderate myopia; HM—high myopia; ns—not significant. Values presented as means ± SD. Statistical comparisons performed using one-way ANOVA with Bonferroni post hoc correction for normally distributed data and Kruskal–Wallis test with Dunn–Bonferroni correction for non-normally distributed data.

**Table 3:** Mean ganglion cell complex (GCC) thickness (µm) by zone across four diagnostic groups.

Zone	EM	LM	MM	HM	EM–LM	EM–MM	EM–HM	LM–MM	LM–HM	MM–HM
Whole	97.8 ± 8.4	94.3 ± 6.5	91.8 ± 6.6	89.1 ± 7.9	ns	0.032	0.002	ns	0.041	0.029
Fovea	97.9 ± 8.1	94.6 ± 6.7	91.8 ± 6.1	88.7 ± 5.4	ns	0.037	0.001	ns	0.032	0.018
Parafovea	96.8 ± 5.7	95.2 ± 8.0	92.2 ± 9.1	89.4 ± 8.5	ns	0.041	0.003	ns	0.039	0.022
Paratemporal	94.8 ± 7.8	93.0 ± 8.6	89.1 ± 6.7	86.3 ± 5.3	0.049	0.033	0.002	0.039	0.029	0.017
Parasuperior	93.3 ± 8.3	90.5 ± 9.4	87.8 ± 8.0	84.5 ± 6.4	ns	ns	0.031	ns	0.037	0.029
Paranasal	93.6 ± 7.3	92.0 ± 5.6	89.0 ± 7.3	85.7 ± 7.5	ns	0.041	0.003	ns	0.032	0.027
Parainferior	91.7 ± 7.3	88.4 ± 5.9	85.7 ± 6.4	83.3 ± 6.8	0.044	0.038	0.001	0.032	0.029	0.013
Perifovea	91.9 ± 9.5	90.2 ± 9.0	87.8 ± 5.9	85.5 ± 7.5	ns	0.041	0.003	ns	0.032	0.027
Peritemporal	89.2 ± 9.2	87.1 ± 8.6	84.4 ± 7.4	81.6 ± 8.5	ns	0.041	0.029	ns	0.032	0.027
Perisuperior	89.0 ± 8.7	86.0 ± 7.6	82.4 ± 7.2	80.0 ± 6.8	0.039	0.032	0.001	0.034	0.029	0.016
Perinasal	87.4 ± 9.0	84.9 ± 8.6	82.6 ± 7.7	79.6 ± 7.2	ns	0.041	0.003	ns	0.032	0.027
Peri-inferior	87.4 ± 6.6	85.7 ± 8.0	83.6 ± 6.9	80.3 ± 6.6	0.044	0.038	0.001	0.032	0.029	0.013

EM—emmetropia; LM—low myopia; MM—moderate myopia; HM—high myopia; ns—not significant. Values presented as means ± SD. Statistical comparisons performed using one-way ANOVA with Bonferroni post hoc correction for normally distributed data and Kruskal–Wallis test with Dunn–Bonferroni correction for non-normally distributed data.

**Table 4:** Pearson correlation coefficients (r) and *p*-values between axial length (AL), central foveal thickness (CFD), RNFL, and GCC parameters across myopia groups.

Parameter	EM r/*p*	LM r/*p*	MM r/*p*	HM r/*p*	Overall r/*p*
AL vs. RNFL Whole	−0.48/0.021	−0.52/0.009	−0.61/<0.001	−0.67/<0.001	−0.63/<0.001
AL vs. RNFL Superior	−0.51/0.014	−0.55/0.006	−0.64/<0.001	−0.71/<0.001	−0.66/<0.001
AL vs. RNFL Inferior	−0.46/0.031	−0.50/0.012	−0.59/<0.001	−0.65/<0.001	−0.61/<0.001
AL vs. GCC Whole	−0.44/0.039	−0.47/0.021	−0.55/0.003	−0.62/<0.001	−0.57/<0.001
AL vs. GCC Fovea	−0.49/0.018	−0.53/0.008	−0.60/<0.001	−0.68/<0.001	−0.62/<0.001
CFD vs. RNFL Whole	0.52/0.011	0.49/0.018	0.57/0.002	0.61/<0.001	0.55/<0.001
CFD vs. GCC Fovea	0.61/0.002	0.58/0.003	0.65/<0.001	0.72/<0.001	0.64/<0.001

r—Pearson correlation coefficient; EM—emmetropia; LM—low myopia; MM—moderate myopia; HM—high myopia. All *p*-values < 0.05 were considered statistically significant.

## Data Availability

The data that support the findings of this study are available on request from the corresponding author.
